# Zebrafish as an Animal Model for Testing Agents with Antidepressant Potential

**DOI:** 10.3390/life11080792

**Published:** 2021-08-05

**Authors:** Joanna Lachowicz, Karolina Niedziałek, Ewelina Rostkowska, Aleksandra Szopa, Katarzyna Świąder, Jarosław Szponar, Anna Serefko

**Affiliations:** 1 Student’s Scientific Circle at Laboratory of Preclinical Testing, Medical University of Lublin, Chodźki 1, 20-093 Lublin, Poland; 54197@student.umlub.pl (J.L.); 56008@student.umlub.pl (K.N.); 2 Beauty Salon “Clarie Beauty”, Pułaskiego 26, 20-461 Lublin, Poland; farm.stos@umlub.pl; 3 Laboratory of Preclinical Testing, Chair and Department of Applied and Social Pharmacy, Medical University of Lublin, Chodźki 1, 20-093 Lublin, Poland; 4 Chair and Department of Applied and Social Pharmacy, Medical University of Lublin, Chodźki 1, 20-093 Lublin, Poland; katarzyna.swiader@umlub.pl; 5 Clinical Department of Toxicology and Cardiology, Medical University of Lublin, Chodźki 1, 20-093 Lublin, Poland; jaroslawszponar@umlub.pl; 6 Toxicology Clinic, Stefan Wyszyński Regional Specialist Hospital in Lublin, Al. Kraśnicka 100, 20-718 Lublin, Poland

**Keywords:** zebrafish, *Danio rerio*, antidepressants, new animal model, screening research

## Abstract

Depression is a serious mental disease that, according to statistics, affects 320 million people worldwide. Additionally, a current situation related to the COVID-19 pandemic has led to a significant deterioration of mental health in people around the world. So far, rodents have been treated as basic animal models used in studies on this disease, but in recent years, *Danio rerio* has emerged as a new organism that might serve well in preclinical experiments. Zebrafish have a lot of advantages, such as a quick reproductive cycle, transparent body during the early developmental stages, high genetic and physiological homology to humans, and low costs of maintenance. Here, we discuss the potential of the zebrafish model to be used in behavioral studies focused on testing agents with antidepressant potential.

## 1. Introduction

Depression is a serious mental disease that, according to statistics, affects 320 million people worldwide. The World Health Organization indicates that depression is a leading cause of disability and suicide globally [1]. Additionally, a current situation related to the COVID-19 pandemic has led to a significant deterioration of mental health in people around the world. Recent studies on the influence of COVID-19 on the psychological state in the general population carried out by Xiong et al. [2] revealed that depressive symptoms were reported by 14.6–48.3% of respondents.

Pharmacotherapy is the main treatment strategy for depressive disorders. Despite access to a wide variety of drugs, many patients do not respond properly to the first-line treatment and do not achieve complete remission [3]. As the commonly used antidepressants are inefficient in 50% of patients, finding a good treatment option for all patients is a real challenge. Moreover, treatment-resistant depression may affect up to 1/3 of depressive patients [4,5]. Additionally, given the complexity of both the disease and its causes, development of new antidepressants is a big problem. Novel substances should be more effective, have a faster onset of action, and, at the same time, they should exert fewer side effects, so that they are better tolerated by patients.

Rodents have a long history among the animal models used to study depression [6,7]. In experimental pharmacology, animals are applied to study the neurobiological basis of depression, identify molecular mechanisms of action of antidepressant drugs, and to search for new compounds. However, the use of animal models is associated with limitations, including impossibility to capture all of the phenotypic features of depression in an animal subject (such as sadness, guilt, or suicidal thoughts) [8]. Additionally, ethical dilemmas occur in relation to some methods used to induce depressive symptoms. Therefore, new testing solutions that can be applied in studies focused on depression are being searched for all the time.

In recent years, *Danio rerio* (zebrafish) has emerged as a new model organism that might serve well in preclinical studies, which has been confirmed by a significant increase in a number of publications about this species [9]. Zebrafish have also been used in studies on central nervous system (CNS) diseases, including mood disorders. In this article, we outline the benefits and potential use of *Danio rerio* in depression research (including depression co-existing with anxiety), as well as limitations associated with this animal model. The review also summarizes results of recent studies on commonly administered antidepressants carried out on this fish species.

## 2. *Danio rerio* Characteristics

*Danio rerio*, also named zebrafish, are small, transparent freshwater fish whose standard length is about 40 mm. They have a clear-colored body with parallel black stripes running along it [10]. The shape of their body is fusiform, laterally flattened with eyes centrally located. The zebrafish caudal fin is striped, whereas their dorsal fin has a navy blue area [11]. Currently, zebrafish very often serve as a model organism in genetic and embryonic research on vertebrate development [10]. The growth of the respective zebrafish stages occurs very fast [12]. It begins with a newly fertilized cell that starts to divide. A zygote, blastula, and gastrula with the embryonic axis develop consecutively. After 10 h, the embryo forms somites, a pharyngeal arch, neuromers, and a tail. Organogenesis begins with the onset of development of all basic organs. At this stage, the earliest movements can be detectable and the ear with two otoliths are noticeable [13,14]. After 24 h, the embryo is in the phylotypic phase and its curved body axis starts to straighten up. Furthermore, the development of pigmentation, fins, and blood circulation begins, and the heartbeat can be recorded [14]. After 48 h, rapid morphogenesis is completed, as well as the development of the head, chest, main internal organs, and cartilage [14]. Whereas most of the zebrafish internal organs are formed within 72 h post fertilization (hpf) [15], their digestive system develops within the first 5 days of life. During this time period, malformations can also occur, either naturally or as a result of exposure to toxins [12]. Not all *Danio rerio* embryos develop at the same pace, despite being exposed to the same environmental conditions. Furthermore, the ambient temperature, number of fish in the tank, and water quality have an impact on the development rate [14]. The embryonic period associated with a rapid morphological development ends when a larva has hatched from an egg, and it usually occurs on the third or on the fourth day after fertilization. After that, the development of a larval form begins, which lasts about 6 weeks. During this period, fish triple their length and undergo several morphological transformations, including modifications of their fins and body pigmentation, and they start to more closely resemble a juvenile form. The juvenile form of *Danio rerio* is very similar to the adult form, except for the presence (or absence) of the larval fin fold (present in larvae) and scales (present in adults) [16]. Researchers successfully sequenced the zebrafish genome a few years ago [10]. It has been demonstrated that the zebrafish genome contains 25 chromosomes, with genes highly similar to the human ones. Moreover, it has been revealed that about 70% of human genes have their analogues in *Danio rerio* [12]. 

A great advantage of zebrafish as a research model is its small size, body transparency, and a low-cost husbandry and breeding of zebrafish [15]. It is possible to keep a large number of these small animals in a relatively limited area [17]. The aquatic environment in which they live makes it possible to dissolve tested substances either in the E3 medium (when experiments are carried out in embryos and young larvae) or in the systemic water (when experiments are carried out in older subjects), and thus, zebrafish can absorb them freely [17]. Another advantage is the high fertility rate of *Danio rerio*, in that they lay eggs in large numbers, about 200 at a time. Furthermore, eggs are transparent and their development takes place outside the female’s body. Therefore, it is possible to easily observe zebrafish growth for the first few days of their life under a microscope, modify their development, and monitor their internal organs [10]. The laid zebrafish eggs can be fertilized in vitro and the zebrafish sperm can be frozen [18]. Typically, the growth and maturation period from an embryo to an adult form lasts about 3 months. Thanks to the rapid development of *Danio rerio*, multigenerational studies can be easily designed. At last, *Danio rerio* as an animal model poses lesser ethical issues in comparison to rodents [10]. 

*Danio rerio* have been used more and more frequently in biomedical studies [17]. They play an important role in several branches of biomedical science, including molecular biology, cardiology, neurology, ophthalmology, oncology, genetics, embryonic research, or environmental toxicology [10,12,17]. The zebrafish genome is sensitive to manipulations, including fluorescent labeling [15], and thus, it is possible to induce genetic changes that affect fish development, organogenesis, and physiology [19]. Furthermore, genetic manipulations open up an opportunity to induce diseases, such as Huntington’s disease [20], Alzheimer’s disease [21,22], sideroblastic anemia [17], attention-deficit hyperactivity disorder [23], diabetes and obesity [24,25], osteoporosis [26], and cardiovascular diseases such as arrhythmia [27,28]. At last, cancer cells can be transplanted into the zebrafish body in order to observe stages of tumor development and the metastatic process [10].

In the following sections of our review, we will focus on the possible applications of zebrafish in neurological research, especially as a model to evaluate CNS disorders, including depression.

## 3. Zebrafish in Neurological Studies

Modeling of CNS diseases involves significant problems, including poor understanding of some of their mechanisms or overlapping symptoms of various diseases. Additionally, it is not possible to fully design a given human neurological/mental disease, with its complexity, in animals. Thus, when carrying out pre-clinical experiments researchers usually focus only on the selected phenotypic features of a given syndrome/illness. In laboratory animals with induced depression-like behavior, it is not possible to assess guilt or suicidal thoughts, which are common (and serious) symptoms in depressive people that should be particularly alleviated/treated. Thus, this methodological deficiency can negatively influence the obtained results and may complicate, or even falsify, their interpretation. Three criteria have been defined for animal models used for studying depression: (1) predictive validity, (2) face validity, and (3) construct validity [29]. The first criterion requires that an animal model should respond to conventional antidepressant drugs in a specific and selective manner, in a similar way that is observed in the human population. The face validity assumes that symptoms of induced depression-like behavior in animals are similar to those observed in humans, including similarity of biochemical changes [30]. The last requirement, i.e., the construct validity, has various definitions, but its point is that the etiology of specific symptoms of depression in animal models and humans should be similar. This criterion takes into account a theoretical approach to the development of depressive disorders [29,31]. Long-term preliminary studies on the zebrafish model have revealed that it is possible to use *Danio rerio* in neurobiological research for designing in vivo models of human diseases associated with disturbed social behavior, including depression and anxiety (which will be described in the next sections of the present paper), but also autism [32,33], obsessive compulsive disorder [34], attention deficit hyperactivity disorder (ADHD) [35,36], addictions [37,38,39], or Alzheimer’s disease [40,41].

Zebrafish, with well-defined and well-established behavior [42,43], present a whole range of social interactions, since they live in groups (shoals) in their natural environment. Complex social behaviors, that include spatial preference for conspecifics, orienting behavior, shoaling, or schooling, are acquired by this species fairly quickly and they develop between the 10th and the 16th day post fertilization (dpf). Additionally, homologous structures and/or regions with analogous functions responsible for social behavior have been identified in the brains of mammals and *Danio rerio* [44,45]. Furthermore, zebrafish, being animals active during the daytime, respond to visual stimuli in a way more similar to humans than rodents do [33]. Zebrafish synthesize the most significant (with respect to human organisms) neurotransmitters [46] and possess brain regions/structures that functionally correspond to regions/structures in the human brain, including the hippocampus and amygdala, which play an important role in CNS diseases [47,48]. Due to the fact that *Danio rerio* also produce hormones, transporters, and receptors, physiological similarity (to a reasonable degree) between a given neurological disease induced in zebrafish and the same disease developed in humans can be achieved [49].

An important advantage of zebrafish is the possibility to select subjects at the most appropriate stage of development for a given experiment. For example, *Danio rerio* larvae have transparent bodies which makes it possible to observe the development of the brain and to visualize specific neurons [50]. On the other hand, adult fish with more complex behavior patterns and with developed motor, sensory, and endocrine systems offer greater possibilities to design disease phenotypes [51]. A well-described zebrafish genome along with the relative ease of introducing genetic manipulations into the *Danio rerio* genome open up opportunities to create transgenic models with CNS diseases. It has been suggested that the development of zebrafish transgenic lines requires only 1/3 of the time needed to obtain a similar transgenic rodent line [34], which significantly reduces the duration of experiments and as a consequence, considerably diminishes costs.

### 3.1. Studying Depression-Like Behavior in Zebrafish

#### 3.1.1. Behavioral Tests

Principal tests, well-described in the scientific literature, used in experiments in zebrafish focused on affective/anxiety disorders (including depression) are partly analogous to those used in rodent models. Animal models of depression should fulfill construct validity. Therefore, in rodents, most of them have been developed on the basis of stress exposure (acute or chronic stress) or exogenous administration of glucocorticoids. It should be remembered that the application of stress to rodents also produces anxiety-like responses. Similarly, anxiety and depression often overlap clinically. In animal models, the distinction between stress-induced depression-like and anxiety-like behaviors is difficult to determine, particularly since both types of behaviors respond to antidepressants [8].

One of the most frequently used tests is the novel tank test (NTT), in which adult *Danio rerio* subjects are placed in a rectangular tank, divided into two equal horizontal parts. According to the test protocol, fish should be allowed to swim freely for 5–30 min. In the NTT, alterations in the behavior pattern of zebrafish are detected. Normally, when put in a new environment, *Danio rerio* initially spend most of the time in the “protection zone” (i.e., in a lower part of the tank), and after they have acclimatized to the new place, they begin exploring it. The most important parameters recorded in the NTT that describe the anxiety-/depression-like behavior of tested subjects are the time spent in the upper and lower parts of the tank, number of entries to the upper part, delay in entering the upper part, freezing time, irregular movements, immobility, swimming speed, and travelled distance [33,51,52]. Many researchers underline an additional advantage of this test carried out in zebrafish, that is not attributed to experiments in rodents, i.e., the possibility to analyze the animal’s behavior in three dimensions, which provides more relevant data [53,54]. Though the NTT is based on similar assumptions as the open field test (OFT) in rodents, the typical OFT (analogous to the OFT in rodents) can also be carried out in *Danio rerio.* The swimming pattern, exploratory behavior, and general locomotion of *Danio rerio* subjects are analyzed in the zebrafish OFT. Different swimming zones in a tank are usually delineated in order to measure the time spent in a respective area, e.g., at the edge of the tank vs. in the center of the tank. Moreover, the travelled distance, swimming speed, number of freezing episodes, and time of immobility are also evaluated [55,56].

Another behavioral test carried out in zebrafish, that has been adapted from rodents, is the light/dark paradigm. This test is based on the natural conflict between staying in a safe environment (i.e., a dark zone) and exploring a new place (in this case a lighted zone). Generally, adult *Danio rerio* subjects prefer being in a dark environment, but they also present exploratory behavior [57]. The light/dark paradigm is mainly used to evaluate anxiety-related phenotypes, and it is usually applied to determine an anxiolytic or anxiogenic potential of a tested substance. Though the test itself is not designed to assess antidepressant activity, it may be useful in the detection of additional anxiolytic/anxiogenic effects exerted by an agent that possesses an antidepressant potential. In view of the fact that depression very often co-exists with anxiety, such an assessment seems to be very important. For example, clonazepam, a strong anxiolytic benzodiazepine, reduces the time spent in the dark zone by adult *Danio rerio* and increases their locomotor activity, whereas an acute exposure to fluoxetine and imipramine (i.e., conventional antidepressant drugs) prolonged the time spent in the dark environment by zebrafish in experiments carried out by Magno et al. [58], suggesting an anxiogenic activity of these agents after an acute administration. Reported observations were in line with outcomes from studies carried out in rodent models in which animals subjected to fluoxetine treatment presented the anxiety-like behavior. In fact, anxiety and anxiety-related symptoms belong to side effects of fluoxetine [59,60].

The zebrafish tail immobilization (ZTI) test, recently proposed by the research team of Demin [61], is a behavioral test based on assumptions of the tail suspension test and the forced swim test which are widely used in rodents in order to observe despair-like behaviors. According to the test protocol, the lower (tail) part of the fish body is immobilized for 5 min by suspending it vertically in a beaker filled with water. This new method was validated. The authors demonstrated that stressors such as electric shock or alarm pheromones reduced the locomotor activity of individuals, while sertraline and amitriptyline increased the travelled distance and mobility time of fish in the ZTI test. Interestingly, phenazepam (an anxiolytic benzodiazepine drug) induced no significant behavioral changes in this test. Thus, it has been suggested that the ZTI test could be considered as a tool which facilitates distinguishing substances with an antidepressant potential from agents with an anxiolytic activity. However, further research on this subject is needed [61].

The next test well-described in the literature, i.e., the shoaling test, is based on zebrafish social interactions, which were mentioned above in the present paper. In the shoaling test, a group of fish is placed in a large tank, in which the subjects are exposed to pharmacological agents or various stressors. The animals’ behavior, particularly shoal cohesion and the distance between individuals in the shoal, is measured. When a stressor or a potential danger emerge, an increase in shoal cohesion is observed. On the other hand, anxiolytic substances, such as benzodiazepines, weaken these defense mechanisms; shoals become less coherent and individual subjects swim away from the group [56,62,63]. Another test, the social preference test, is also based on the social interactions of fish. More precisely, *Danio rerio* behavior towards their conspecifics is observed in this test. This study consists of two phases: during the first phase, a single subject habituates in a new tank and during the second phase, the so-called interaction phase, a group of fish is introduced into the tank (alternatively, a recorded video can serve as a social stimulus). The principal parameter measured in the social preference test is the time spent by the observed fish in areas close to the social stimulus [64]. Therefore, both the shoaling test and the social preference test could serve as simple tools to investigate mechanisms of neuropsychiatric diseases that are associated with an impairment of social interactions.

Amongst other behavioral tests used in *Danio rerio*, the scientific literature also mentions tests in which zebrafish behavior in the presence of a new object or a predator is observed. This test is usually carried out in an environment well-known for a given subject, and a new object or a natural predator of *Danio rerio* (alternatively, its imitation) are introduced into the tank. Latency to approach a new object, time spent in the vicinity of a new object, number of freezing episodes and their duration, or alternatively, time spent in the vicinity of a predator, latency to enter the zone in which a predator is located, and number of such entries are measured [56]. A list of the behavioral tests discussed above is presented in Table 1. Unfortunately, the results obtained in behavioral tests in zebrafish listed in Table 1 can be affected by several factors, such as genetic background, zebrafish line, sex, age, number of animals in the tank, between-individual variations, between-laboratory variations [65], and others (see the most common limitations mentioned in Table 2).

#### 3.1.2. Different Methods to Induce Depression-Like Behavior in Zebrafish

Reserpine is an antipsychotic drug with an anxiolytic and antihypertensive activity. Its mechanism of action involves an irreversible blockage of the vesicular monoamine transporter which in consequence results in reduced levels of monoamines in the brain. Therefore, one of the significant side effects of reserpine treatment is the development of depressive behavior which has been used in preclinical studies—reserpine serves as an agent that induces depression-like behavior in laboratory animals [94,95]. Though previously reserpine was mainly applied in rodent models, lately it has also been used in *Danio rerio*. After administration of reserpine, zebrafish exhibit hypolocomotion, social withdrawal, and their cortisol levels are elevated [25,96]. Recent studies have shown that sertraline, one of the most commonly used selective serotonin reuptake inhibitors (SSRIs), significantly increased locomotor activity and decreased the reserpine-induced depression symptoms in adult zebrafish [25]. A similar effect was obtained after exposure to venlafaxine, a serotonin-norepinephrine reuptake inhibitor (SNRI). In the studies by Tang and colleagues [97], exposure to venlafaxine reversed the reserpine-induced depressive behavior in adult zebrafish, i.e., it increased exploratory activity (as well as an exploratory behavior) and reduced the frequency of irregular movement or freezing episodes in the NTT. 

Another method used to induce depression-like behavior in zebrafish is the chronic unpredictable stress (CUS) paradigm, which is one of the most widely applied, reliable, and effective models of depression in rodents. It has been demonstrated that after exposure of adult *Danio rerio* to the CUS, significant behavioral and physiological changes (i.e., anxiety-like behavior, increased levels of cortisol and corticotropin-releasing factor, decreased expression of the glucocorticoid receptor) can be observed within 7–14 days of the study. Given the time needed to carry out the CUS protocol in rodents, *Danio rerio* emerges as a much faster alternative [98,99]. In the CUS in zebrafish, stressors are used randomly to avoid habituation. Temperature changes (heating up to 33 °C or cooling down to 23 °C), social isolation, crowding a large number of fish in a small tank, lowering the water level in the tank, or multiple tank changes are applied [100]. Additionally, Marcon et al. [100] demonstrated that exposure to the CUS increased the gene expression of pro-inflammatory markers (i.e., interleukin 6 and COX-2) in the zebrafish brain, which in turn indicates that the CUS model in *Danio rerio* has a potential to be useful in studies focused on the inflammatory theory of depression [101]. Zebrafish previously subjected to the CUS protocol presented significantly changed behavior in the NTT, i.e., a reduced number of entries to the upper part of the tank as well as a reduction in time spent in the upper part of the tank were recorded. It was also observed that fluoxetine and nortriptyline prevented the effects induced by the CUS. Furthermore, both of these drugs decreased levels of whole-body cortisol and reduced levels of pro-inflammatory markers (i.e., COX-2 and Il-6) in the zebrafish brain [100].

Transgenic zebrafish are also used in preclinical studies focused on depression. *Danio rerio* with an induced mutation in the gene encoding the glucocorticoid receptor displays depression-like behavior [102]. It has been demonstrated that adult grs^357^ zebrafish mutants behaved differently from their wild-type counterparts in behavioral tests and that their specific behavior could have qualified as a depressive one. On the other hand, zebrafish larvae (5 dpf) with the same mutation showed only changes in their locomotor activity. Most probably, the observed differences in behavior between adult and larval subjects could be attributed to differences in the functional maturity of the hypothalamus–pituitary–interrenal (HPI) axis [103]. The research team led by Griffiths [49] confirmed that zebrafish larvae with grs^357^ mutation display a reduced spontaneous swimming activity. Furthermore, the authors showed that a 24 h exposure to fluoxetine is able to reverse this effect. A long-term treatment with fluoxetine also restored normal behavior patterns in adult grs^357^ zebrafish mutants subjected to the NTT in experiments carried out by Ziv et al. [102]. However, a partial reduction in cortisol levels in the tested subjects was detected only after chronic exposure to fluoxetine [102]. In fact, genetic modifications of the glucocorticoid receptor have already drawn attention when studying depression in mice and rats [104,105]. In rodents, they lead to dysregulation of the hypothalamic–pituitary–adrenal (HPA) axis and to uncontrolled release of cortisol, which ultimately, may cause depression [106].

#### 3.1.3. Toxicological Studies of Antidepressant Drugs

Over the past twenty years, *Danio rerio* has been widely used in ecotoxicology studies. Observation of fish enables determination of an influence of different kind of pollutants on the environment or to evaluate toxic and/or teratogenic effects of xenobiotics [107,108]. A standard protocol used for toxicity assessment is called the fish embryo acute toxicity (FET) test, which was described by the Organization for Economic Co-operation and Development (OECD) as test guideline No. 236. This test makes it possible to estimate the mortality rate of *Danio rerio* and to observe developmental disorders during the first five days of their life [109]. Carrying out experiments in a zebrafish model, it is possible to characterize, inter alia, the neurotoxicity of a given substance by simply analyzing swimming activity/swimming pattern of tested larvae, or by evaluating more complex behavior of adult fish, such as learning ability or social interactions [50,110]. It turned out that *Danio rerio* can be useful in the toxicological assessment of substances with an antidepressant potential. In fact, antidepressant drugs pose a significant problem to the environment as environmental pollutants. Due to their mechanism of action, they modulate the activity of neurotransmitters, and it is assumed that in consequence they may influence different biological processes in living organisms exposed to their residues in the water system by disturbing their behavior, reproduction, and development [111]. The zebrafish model has been used in ecotoxicological studies of antidepressant drugs that were tested at concentrations detected in the aquatic environment. It has been revealed that sertraline, when tested at low (environmental) concentrations, accelerated the hatching process of *Danio rerio* larvae (at a dose of 100 μg/L), produced changes in *serta* and *5-ht2c* expression (at a dose of 10 μg/L), and induced altered behavior, such as avoiding dark zones of a tank (at doses of 1–100 μg/L) [112]. Similarly, fluoxetine at doses detected in municipal water samples induced hypolocomotion in zebrafish larvae and delayed their hatching. In addition, a dose-dependent inhibitory effect on acetylcholinesterase (AChE) activity was observed in the tested subjects [113]. On the other hand, similar doses of citalopram, another SSRI, did not lead to any significant changes in the swimming behavior of the tested larvae as compared to the control group [114]. However, studies by Tang and colleagues [115] demonstrated that the environmental concentrations of venlafaxine had a dual effect on the activity of *Danio rerio* larvae—at higher tested concentrations it reduced the mobility of zebrafish, while the lowest tested concentrations induced hyperlocomotion. The results of these experiments carried out in zebrafish also allow us to suggest that the mechanism of toxic action of venlafaxine may be associated with upregulation of the cAMP–pCREB pathway, which may also increase cortisol levels [115].

The zebrafish model is well suited for screening the biological activity of different compounds, including their toxicity [116,117]. It can provide a basis for assessing the risk associated with administration of a given agent to humans and it can reveal a potential developmental toxicity due to similarity of developmental pathways between zebrafish and mammalians [118]. However, it should be remembered that the toxicology research with *Danio rerio* provides only preliminary results on the toxicity/safety profile of tested compounds, therefore they cannot be directly translated into clinical practice and require confirmation in studies carried out on other animal models, e.g., rodents, dogs, and/or primates.

### 3.2. Limitations of Zebrafish Models 

Apart from multiple advantages, the zebrafish model has its limitations and disadvantages, including a shortage of well-characterized inbred strains and a paucity of mutants [119]. However, thanks to an increasing interest in zebrafish models, these problems will most probably be solved in few years. Some behavioral patterns, such as social behaviors, develop rather late in zebrafish life, so it is not possible to study them during the early larval stages [120]. The brain of *Danio rerio* lacks several structures that exist in the mammalian brain. Though their functions usually are taken over by subcortical centers [47,48], interpretation of the obtained outcomes can be difficult in some specific studies. An obvious advantage of experiments in zebrafish is the possibility to dissolve a tested substance in the tank water, which eliminates the stress induced by an injection and its potential influence on the obtained results [121]. However, a problem emerges when an insoluble compound is going to be tested. Certainly, it is possible to add a cosolvent, but both ethanol and dimethyl sulfoxide (DMSO) can have a negative effect on the development of *Danio rerio* in the early stages of their life [122,123]. There are also reports on significant differences in behavioral patterns between distinct zebrafish strains as well as between the sexes. *Danio rerio* females show higher dopamine levels and a lower 5-hydroxyindole acetic acid to serotonin ratio when compared to males [124]. Dahlbom and colleagues [124] found out that female and male zebrafish subjects differ in terms of how they react to stressors. The authors demonstrated that males are more stressed than females. Another research team [125] revealed that the CUS protocol induced a higher level of aggressive behavior in males when compared to females. Furthermore, male subjects presented higher cortisol levels after exposure to stress than female ones. On the one hand, this feature opens up the possibility to study differences between the sexes in depression, which are also known in humans [126]. On the other hand, this feature poses a significant methodological problem when designing a study, since sex determination in *Danio rerio* takes place ca. 20–25 dpf [127]. Furthermore, the latest experiments by Cui et al. [128] demonstrated the sex-specific accumulation of fluoxetine in adult zebrafish when exposed to the drug dissolved in aquarium water. Considerably higher concentrations of fluoxetine were observed in females as compared to males. Similarly, sexual differences were observed in relation to alteration in the brain serotoninergic system induced by the prolonged exposure to fluoxetine. Females presented changes in the serotonin transporter (SERT) along with disturbances in the serotonin release, whereas males displayed alterations in brain levels of tryptophan hydroxylase which negatively influence synthesis of serotonin. After fluoxetine exposure a more profound increase in monoamine oxidase activity was detected in males when compared to females. Vera-Chang and colleagues [129] highlighted the importance of both sex- and time-dependent differences in response to antidepressant treatment in zebrafish. They found out that adult female zebrafish are more sensitive to the effects of fluoxetine when exposed to the drug between the 15th and the 42nd dpf (i.e., during the period of late sexual development)—they presented a considerable level of the exploratory behavior, whereas adult male zebrafish are more sensitive to the effects of fluoxetine when exposed to the drug during the period of early sexual development (i.e., up to the 15th dpf)—they displayed reduced activity. The results obtained by Nielsen et al. [130] also suggest that selective serotonin reuptake inhibitors may induce the sex-specific swimming behaviors in zebrafish. According to the literature data [124,131], a sex-dependent behavioral response in zebrafish can be associated with the sex-dependent levels of androgens and estrogens as well as with the sex-dependent differences in serotonin production. It should be mentioned that both sex- and age-dependent alterations in the antidepressant-like behavior induced by fluoxetine have been detected in rodents [132]. It has been also demonstrated that distinct zebrafish strains display significant differences in manifestation of anxiety-like behavior. Some mutant strains of zebrafish (such as leopard or albino) produce a stronger anxiety-like response when compared to wild-type strains [42]. The above-mentioned aspects should be taken into consideration when designing an experiment with use of the *Danio rerio* model, as well as when translating study outcomes into rodent subjects.

It seems that the most important problem that emerges when studying depression in *Danio rerio* is the inability to appropriately distinguish differences between the locomotor inactivity and the anxiety-like from the depressive-like state. As indicated in the review by de Abreu et al. [133], a more detailed analysis of fish behavior during a given experiment is necessary. As the authors highlighted, in order to correctly classify the depressive- and anxiety-like behavior of zebrafish in the behavioral tests described above, many parameters must be taken into account at the same time. In order to increase the certainty of whether the depressive-, anxiety-, or depressive-anxiety-like behavior have occurred in *Danio rerio*, it is important to check whether reactions of the tested subjects can be reversed by administration of antidepressant and/or anxiolytic treatment. For example, in the NTT, zebrafish displaying hypolocomotion and maintaining an up/down preference may be treated as subjects with the typical depressive-like behavior, whereas a reduced motor activity accompanied by freezing episodes, irregular movements, and/or a preference for swimming in a lower part of the tank may be specific to the anxiety-like behavior [133]. However, as indicated by de Abreu et al. [133], this cannot be taken for granted. Therefore, it seems a good solution to check whether the observed behavioral changes in fish improve after introduction of conventional antidepressants and/or anxiolytics. If zebrafish behavior is changed by administration of an antidepressant drug, but it remains insensitive to anxiolytic therapy, this may indicate locomotor retardation, which is a characteristic symptom of depression. On the other hand, if *Danio rerio* behaviors, such as limited swimming, frequent freezing states, and irregular movements, are reduced after the administration of anxiolytics and/or may be enhanced by stressful stimuli, it suggests anxiety rather than depression. However, if changes in these fish behaviors are improved after both antidepressants and anxiolytics, it indicates a coexistence of depression and anxiety [133].

In order to better analyze the obtained results, researchers assess levels of biomarkers relevant to depression and/or anxiety. Usually, cortisol levels are measured in the zebrafish body, which is correlated with the stress level [134,135]. Additionally, levels of ACTH (adrenocorticotropin) and POMC (pro-opiomelanocortin), which are also involved in the physiological response to stress, can be determined [136,137]. As widely indicated in both preclinical as well as clinical studies, the hypothalamic–pituitary–adrenal (HPA) axis is engaged in the body’s response to acute and chronic stress. After introduction of a given stimulus, the endocrine cascade for stress responsivity is activated. There is an increase in the corticotrophin-releasing hormone (CRH), POMC, ACTH, and cortisol (named as “the stress hormone”) levels [138]. Moreover, it was demonstrated that these hormones are closely associated with specific behaviors, such as anhedonia, depression- and anxiety-like behavior, and cognitive deficits, both in animals and in humans [139,140,141]. Consequently, levels of CRH, POMC, ACTH, and cortisol could be treated as biomarkers with potential translational use in depression and anxiety [142]. A relationship between CRH, ACTH, and cortisol concentrations and treatment outcomes in depression has been demonstrated (for review see [143]). Knowing the molecular mechanism of action of the applied antidepressant agent, it is possible to determine its influence on the level of a respective monoamine neurotransmitter in the brain, which can also be affected by exposure to a stressor [144,145]. When analyzing the stress-related behavior in zebrafish, expression levels of pro-inflammatory markers are assessed as well [146,147].

## 4. Summary

To summarize, this paper presents a whole range of possibilities created by the zebrafish model in preclinical studies focused on depression—from behavioral testing to designing genetic mutants. Multiples studies carried out in recent years, listed in Table 3, have confirmed that *Danio rerio* are sensitive to conventional clinically used antidepressant drugs. Since these well-known agents are able to reverse a depressive phenotype in zebrafish, it allows us to suggest that the discussed animal model may be useful in the search for new molecules with antidepressant and/or anxiolytic potential. In addition, experiments in the *Danio rerio* model follow the 3R principle (replacement, reduction, and refinement), which aims to reduce the number of animals used in preclinical studies as well as to reduce their suffering [148]. Despite certain limitations, the zebrafish model offers an interesting alternative in the field of neurobiological research. *Danio rerio* can be used by scientists for high-throughput screening of novel molecules and should be treated as a supplementary tool to the existing methods applied in experimental pharmacology.

## Figures and Tables

**Table 1:** Behavioral tests used in depression-/anxiety-like behavior assessment in zebrafish.

Behavioral Test	Test Description	Ref.
Novel tank test (NTT)	Similar to the OFT in rodents. Zebrafish are placed in a tank, divided into two equal horizontal parts and the following parameters are measured: the time spent in upper and lower parts of the tank, number of entries to the upper part, latency to enter the upper part, freezing time, irregular movements, immobility, swimming speed, and travelled distance.	[33,51,52]
Open field test (OFT)	Similar to the OFT in rodents. Zebrafish are placed in a novel tank and the following parameters are measured: thigmotaxis (peripheral swimming) and exploration behavior.	[55,56,66]
Light/dark test	Similar to the light/dark test in rodents. The test evaluates scototaxis (preference for dark environments) by recording latency to enter a white half of the tank, time spent in the white half, and number of entries to the white half.	[58,67,68]
Zebrafish tail immobilization test (ZTI)	Similar to the forced swim test and the tail suspension test in rodents, which detect the despair-like phenotype. The most important parameters are overall activity, overall mobility, time spent mobile or active, and number of activity episodes.	[61]
Shoaling test	Similar to the social behavior test in rodents. The test measures an influence of the anxious state on social interactions. The most important parameters are distance between subjects and height of exploration.	[56,62,63]
Social preference test	Similar to the social behavior test in rodents. In this test, *Danio rerio* behavior towards their conspecifics is observed. The time spent by the observed fish in the areas proximal to the social stimulus is measured.	[64]
Novel object approaching	Similar to novelty exposure task in rodents. In this test a novel object is introduced into a tank well-known for a given subject, and the following parameters are measured: latency to approach to a new object, time spent in the vicinity of a new object, thigmotaxis, and number of freezing episodes and their duration.	[56,69,70]
Predator avoidance	Partially similar to predator avoidance in rodents. In this test, a natural predator of zebrafish is introduced into a tank well known for a given subject, and the following parameters related to avoidance and fear are measured: distance between the predator and a tested subject, geotaxis (diving towards the bottom of the tank), activity (i.e., average speed), turn angle, number of freezing episodes, and time spent frozen.	[56,71]

**Table 3:** Antidepressant drugs tested in zebrafish.

Antidepressant Drug	Tested Dose and Duration of Treatment	Tested Zebrafish	Applied Test	Effects	Ref.
Amitriptyline	10 and 50 μg/L; 2-week exposure	Adult subjects	NTT	50 μg/L: anxiolytic-like behavior, i.e., ↑ time spent in the upper part of the tank, ↓ transitions to the upper part of the tank, ↓ latency to enter the upper part of the tank; both doses: ↓ serotonin turnover and ↑ norepinephrine and dopamine levels in the brain	[149]
	1, 5, and 10 mg/L; 20 min exposure	Adult subjects	NTT	1 and 5 mg/L: changes in behavior, i.e., ↑ time spent in the upper part of the tank, ↓ latency to enter the upper part of the tank; 10 mg/L: ataxic movements; 5 and 10 mg/L: ↑ 5-HIAA (5-hydroxyindoleacetic acid)/serotonin ratio in the brain	[150]
	1 mg/L; 20 min exposure	Adult subjects	ZTI	1 mg/L: ↑ travelled distance and ↑ time of mobility	[61]
Escitalopram	0.15 and 1.5 µg/L; 3-week exposure	Adult subjects	Spontaneous swimming behavior	Gender differences in behavioral responses: 1.5 µg/L in females: ↓ maximum swimming velocity and ↓ thigmotaxis; 1.5 µg/L in males: ↓ maximum swimming velocity	[151]
Fluoxetine	0.01 mg/L; 7-day exposure	Adult subjects	CUS + NTT	In stressed subjects: restored swimming in the upper part of the tank, prevented increase in the cortisol level	[100]
	4.6 μM; 24 h exposure	Larvae with grs^357^ mutation	Spontaneous activity	↑ activity and ↓ the whole-body cortisol level	[49]
	0.8 μM; 4-day exposure	Adult subjects with grs^357^ mutation	NTT	↓ freezing duration and ↓ wall-avoidance behavior	[102]
	0.25 mg/L; 3 h exposure	Adult subjects	NTT	↑ time spent in upper part of the tank and ↓ freezing duration	[152]
	10 mg/L; 3 min exposure	Adult subjects	Open-field drop test	↓ bottom dwelling and ↑ time spent in the upper part of the tank	[153]
Nortriptyline	0.01 mg/L; 7-day exposure	Adult subjects	CUS + NTT	In stressed subjects: restored swimming in the upper part of the tank, prevented increase in the cortisol level	[100]
Sertraline	2, 10, and 15 mg/L; 20 min exposure	Adult subjects	ZTI	10 and 15 mg/L: ↑ travelled distance 15 mg/L: ↑ mobility time	[61]
Venlafaxine + Melatonin	0.025 μg/mL + 1 μM; 7-day exposure	Adult subjects	Reserpine-induced depression (40 μg/mL, 7 days) + NTT	Reversed the reserpine-induced depressive phenotype, i.e., ↑ exploration, ↓ irregular movements, ↑ travelled distance in the upper part of the tank	[97]

CUS—chronic unpredictable stress; NTT—novel tank test; ZTI—zebrafish tail immobilization.; ↑: increase; ↓: decrease

**Table 2:** Characteristics and limitations of behavioral tests used in depression-/anxiety-like behavior assessment in zebrafish.

Behavioral Test	Characteristics	Limitations	Translational Impact	Ref.
Novel tank test (NTT)	Until acclimatization to the other parts of the tank, adult zebrafish stay at the bottom of the tank, and then their vertical activity gradually increases over time. Indicators of anxiety-like behavior: the degree of “bottom dwelling”, restriction of exploratory behavior and timotaxia.	(1) Non-standardized conditions of the test (shape and size of the tank, duration of the assay, illumination), method of quantification, and definitions; (2) the depth stimuli may exert the “conditional” influence on anxiety but not the “causal” one; (3) should be carried out along with the light/dark test. Results can be influenced by size of the tank, subjects age, sex, domestication, and duration of an experiment.	Thigmotaxis has been interpreted as anxiety-like behavior in rodents, and time spent on the bottom for zebrafish can be interpreted as thigmotaxis; hence, a measure of anxiety-like behavior.	[52,72,73]
Open field test (OFT)	Zebrafish spend most of their time in the outer zone of the open field apparatus, avoiding the central arena (thigmotaxis). Indicators of anxiety-like behavior: avoidance of the center of an arena.	(1) Fish locomotion adjusted to the size of the arena; (2) freezing (stopping) may be not only the manifestation of anxiety but also a part of decision making, information gathering, and risk assessment behavior; (3) unfamiliar environment can evoke anxiety-like behavioral responses. Results can be influenced by variations in the testing tank parameters (i.e., transparent/ opaque walls), acute stress prior to the test, differences in locomotor activity of subjects, age.	Thigmotaxis has been interpreted as anxiety-like behavior in rodents, and the preference of zebrafish for the outer zone of the open field apparatus can be interpreted as thigmotaxis; hence, a measure of anxiety-like behavior.	[56,73,74,75]
Light/dark test	The zebrafish is diurnal animal, and thus (usually) prefers to stay in a black chamber over a white one. Indicators of anxiety-like behavior: prolonged time spent in the black chamber, freezing (expected to be increased under aversive conditions), distance to bottom (expected to be decreased under aversive conditions), vertical exploration (expected to be diminished under aversive conditions), erratic movements (expected to be increased under aversive conditions), velocity (expected to be diminished under aversive conditions).	(1) Non-standardized conditions of the test; (2) the black/white stimuli may exert the “causal” influence on anxiety but not the “conditional” one; (3) should be carried out along with the NTT. Results can be influenced by illumination level and type, variations in the testing tank parameters, water depth, use of sand/gravel, presence of dividers between compartments, enclosed compartments, acute stress prior to the test, differences in locomotor activity of subjects, and age.	Scototaxis has been interpreted as anxiety-like behavior in rodents, and preference of zebrafish for the dark chambers can be interpreted as scototaxis; hence, a measure of anxiety-like behavior.	[72,75,76,77,78,79,80]
Shoaling test	Zebrafish is a shoaling fish. Shoaling reduces predation risk and can allow for early detection of an approaching predator. Indicators of anxiolytic-like behavior: tendency to break away from the group. Indicators of antidepressant-like behavior: exploration of the higher zones of the tank.	The side-by-side protocol (when single zebrafish are separated physically in the tank, but they are able to see each other and interact) fails to provide a real interaction (which is influenced by pheromones, amino acids, swimming around each other, physical nudges). Results can be influenced by duration of exposure to a given drug, inter-population differences, sex, age, body size, stripe pattern, shoal size, fin length, pigmentation unfamiliar environment, and aquatic flora.	Clonazepam, bromazepam, and diazepam decrease shoal cohesion, whereas buspirone, fluoxetine, and escitalopram increase fish exploration of higher zones of the tank. In humans, these drugs are commonly used to reduce depressive and anxiety symptoms.	[63,81,82,83,84]
Social preference test	In the habituation phase the tested zebrafish is left alone in a chamber of the test tank, whereas in the second phase it interacts with small groups of live/virtual conspecifics. Indicators of anxiety- and depressive-like behavior: reduced social interactions.	Results can be influenced by specific characteristics of the observed fish (i.e., age, sex, personality), characteristics of the group members (i.e., number, size, sex ratio, phenotype, kinship between individuals, visual characteristics living/computer-animated zebrafish images), early environmental conditions, water temperature, tank volume, test room brightness, size and number of the preference areas, presence of environmental enrichment, and duration of the habituation and interaction phases.	Social preference test in zebrafish is similar to the social interaction tests in rodents that are used to assess social deficits, which are also detected in humans with mental disorders, including depression.	[64,85]
Novel object approaching	Zebrafish is fearful of the novel object and spends time near the wall of the arena (thigmotaxis). Indicators of anxiety-like behavior: decreased time spent in the outer thigmotaxis zone, increased time spent in the central zone near the novel object.	Results can be influenced by age, sex, color, size, shape, and bi-dimensionality of object, individual propensity to approach a novel object, trial duration, and previous experience of the subjects.	Ethanol and scopolamine increased the time spent in the inner zone near the object. In humans, scopolamine is effective at reducing depression and anxiety symptoms. Additionally, acute, but not chronic, consumption of alcoholic beverages at low concentrations relieves stress and/or anxiety in humans.	[38,86,87,88,89]
Predator avoidance	Moving away from the predator is considered as active avoidance, and spending more time on the bottom of the tank is considered as passive avoidance. Indicators of fear/anxiety-like behavior: reduced velocity, increased temporal variability of velocity, increased turn angle, increased temporal variability of turn angle, decreased distance from the bottom, increased erratic movement and jumping (leaping), and increased immobility (freezing) after diving response.	Results can be influenced by context, size of the tank, attack speed and size of the predator, predatory species, proximity to refuges, and engagement in other activities.	Predator avoidance in zebrafish is similar to predator avoidance in rodents. Predator stress is one of the most widely used stressors to induce anxiety- and depression-like behaviors in mice and rats. Exposure to predators can induce stress in rodents that is comparable to chronic stress in humans.	[90,91,92,93]
